# Effects of pH and Electrolytes on Castor Oil Emulsions with Various Stabilisers Using *Khaya senegalensis* Gum as an Emulsifier

**DOI:** 10.1155/2021/7049332

**Published:** 2021-10-15

**Authors:** Deryl Nii Okantey Kuevi, Noble Kuntworbe, Enoch Ayertey

**Affiliations:** ^1^Council for Scientific and Industrial Research (CSIR), Water Research Institute, Biomedical and Public Health Research Unit, P.O. Box AH 38, Accra, Ghana; ^2^Department of Pharmaceutics, Kwame Nkrumah University of Science and Technology, Kumasi 00233, Ghana; ^3^Trade Winds Chemist Ltd., P.O. Box 8412, Kumasi, Ghana

## Abstract

Dispersed systems such as emulsions are easily destabilised during processing and storage since they are thermodynamically unstable systems. It is for this reason emulsifiers/stabilisers are frequently employed in pharmaceutical emulsion formulations to increase their short- and long-term kinetic stability. This current study seeks to investigate the potential emulsifying property of gums obtained from *Khaya senegalensis* (family: Meliaceae) trees. Gums were collected, authenticated, oven-dried, milled, filtered, and purified using 96% ethanol. The microbial quality of the gum was assessed following the BP (2013) specifications. The purified gum was free from some selected pathogenic microorganisms, rendering the gum safe for consumption. The emulsifying property was investigated by formulating emulsions using castor oil and employing the dry gum method. The ratios of oil-to-water-to-gum for the formulation of a stable emulsion were determined. The stability of the emulsion was evaluated, and an effort was made to improve the stability by incorporating Tween 80, hydroxypropyl methylcellulose, and xanthan gum. From the results, it can be inferred that Tween 80 (0.5%) was able to stabilise the emulsion. Addition of xanthan gum worsened the creaming. The effects of pH (4.0, 5.5, 7.2, 9.0, and 11.0) and electrolytes (0.1 M of NaCl, KCl, and CaCl_2_) on the physical stability of oil-in-water emulsions were studied during 12 weeks of storage. Percentage creaming volume and whether there was phase inversion were the criteria used as the evaluation parameter. From the percentage creaming volume data, emulsions formulated with both gums showed the lowest creaming volumes at pH of 7.2, followed by the acidic regions (pH 4.0, 5.5), with the basic regions (pH 9.0, 11.0) recording the highest creaming volumes. The effects of the various electrolytes at a constant concentration of 0.1 M on the o/w emulsions were found in this order NaCl < KCl < CaCl_2_. This study proves that *Khaya senegalensis* gum can successfully be employed as an emulsifying agent in pharmaceutical formulations.

## 1. Introduction

Emulsions have been employed for centuries in diverse domains in the pharmaceutical, cosmetic, and food industry [1]. Pharmaceutical emulsions are a class of disperse systems comprising two nonmiscible liquids [2]. The globules of the disperse medium are uniformly distributed in the continuous medium. Since emulsions are a thermodynamically unstable system, a third component, an emulsifier is added to stabilise the system [3]. Emulsifiers reduce the degree of emulsion instabilities such as creaming, coalescence, flocculation, and phase inversion, thereby making the emulsion more consistent [4].

Emulsifiers stabilise the system by forming a thin film around the droplets of dispersed phase [5]. In the pharmaceutical sector, they are formulated and administered orally, topically, and parenterally [4]. Emulsions offer a great variety of benefits such as the ability to deliver both hydrophilic and lipophilic drugs. Again, they are able to deliver drugs that have low permeability to allow for their adequate and reproducible absorption from the gastrointestinal tract (GIT) following oral administration [6]. Despite the significant advantages of emulsions, the potential drawbacks such as maintaining their physical stability cannot be overlooked. It is a very crucial prerequisite that the API in an emulsion be consistently mixed stays physically and chemically stable throughout the shelf life [7]. This is a key in order to dispense a fairly even and precise dose of the drug [8]. Failure of that could lead to inappropriate dosing, which could lead to possible toxicity. All of these limitations are the reasons why they are not extensively employed compared with other dosage forms.

This concern has steered several in the field of research to explore the capabilities of hydrophilic polymers that are obtained from renewable sources since they are ecofriendly, cheap, biodegradable, and compatible to stabilise emulsion globules.

Reportedly, works have proved that for an oil-in-water (o/w) emulsion to be stable, the concentration and type of excipients to be used in preparing the emulsion, more so, the preparation method and storage temperature should be carefully considered [9]. Presently, tragacanth gum is employed in beverages and foods, more importantly in pharmaceutical formulations [10–12]. Galacturonic acid content in tragacanth gum is accountable for its good emulsification properties and ability to form stable emulsions particularly in the acidic region [13]. Nonetheless, there have been several attempts to discover alternative sources of emulsifiers of natural origin; this is because tragacanth gum is quite costly to import. In the quest for different alternatives that are cheaper but possess the same or even better emulsifying properties, *Khaya senegalensis* gum was considered.

*Khaya senegalensis*, commonly known as the African mahogany, from the family Meliaceae, is a tall plant of about 15 m–30 m above the ground and nearly a meter in diameter, which is easily noticed by its ever-green crown. Traditionally, its very bitter bark is employed as a fever remedy and depurative and for the treatment of syphilis. The roots are used for treating sterility, mental illness, and leprosy [14]. The gum has been established to show encouraging properties for its industrial and pharmaceutical uses. *Khaya senegalensis* gum come as long, slender, semitransparent fragments. The gum is identified to comprise exceedingly branched polysaccharides entailing of D-galactose, D-galacturonic acid, 4-O-methyl-D-glucuronic acid, and L-rhamnose [15]. According to Oluwatoyin [16], the gum can be employed as a sustained release agent for tablets for five hours maximum. In addition, the gum is capable of delivering a time-independent release for longer hours when hydroxypropyl methylcellulose (HPMC) is added. Again, the disintegration property of the gum has been evaluated, and the findings were positive [17].

However, studies have not been conducted on *Khaya senegalensis* gum pertaining to its emulsifying capability. Therefore, it is imperative to evaluate its emulsifying property.

## 2. Materials and Methods

### 2.1. Materials

The crude *Khaya senegalensis* gum was collected from a forest area in a town known as Kwahu-Asakraka in the eastern part of Ghana by incision on the trees of *Khaya senegalensis* of the family Meliaceae. The gums were authenticated, oven-dried, milled, filtered, and purified using 96% ethanol. Castor oil, xanthan gum, hydroxypropyl methylcellulose, nutrient agar, MacConkey agar, mannitol salt agar, cetrimide agar, bismuth sulphite agar, and tragacanth gum were obtained from the chemical store of the Department of Pharmaceutics, Kwame Nkrumah University of Science and Technology (KNUST), Kumasi, Ghana.

## 3. Methods

### 3.1. Collection and Authentication of Gum

*Khaya senegalensis* gum was collected from Kwahu-Asakraka in the eastern region of Ghana by incision on the stem bark of the tree. Gums were harvested between July and August 2017. The *Khaya senegalensis* tree was identified and authenticated by the curator of the Herbal Medicine Department of the Faculty of Pharmacy and Pharmaceutical Sciences, KNUST, and a voucher specimen was kept at the herbarium with a voucher number assigned to it.

### 3.2. Preparation and Purification of Gum

**Figure 1:**
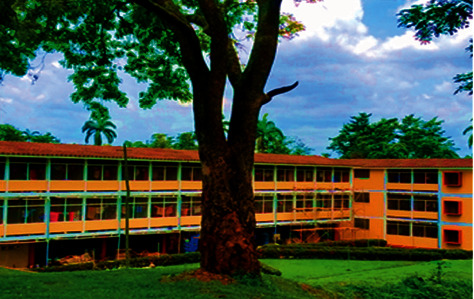
*Khaya senegalensis* tree.

The crude *Khaya senegalensis* gum was obtained by incision as shown in Figure 1 and cleaned by getting rid of the bark and other foreign materials by hand picking, breaking, and sieving. The gum was dried in an oven at 60°C for about 7 hours until it turned out to be adequately brittle. The dried gum was then classified into two shades: light-coloured shade and dark-coloured shade. The light-coloured shade was picked out for further processing by milling in a blender into a fine powder. The powdered gum was utilised as a part of some of the consequent tests and investigations as rough *Khaya senegalensis* gum powder. To filter, 100 g of the crude gum powder was dissolved in 200 mL of distilled water and was permitted to remain for 24 hours. The gum mucilage obtained was filtered using a calico cloth by gripping and pressing firmly to withdraw any insoluble material. The filtered mucilage was refiltered to ensure that all debris was removed. The filtrate was precipitated with three times the volume of 96% ethanol to obtain the purified gum, which was then filtered and washed with diethyl ether. The gum was subsequently dried in a hot air oven at 40°C for 24 hours [18]. The dried purified gum was milled and sifted through sieve number 80. The powdered gum was packed in an airtight container and stored in a desiccator.

### 3.3. Physicochemical Properties of Gum

#### 3.3.1. Extracted Gum Yield

The dried extracted *Khaya senegalensis* gum was weighed, and the percentage yield expressed comparatively to the initial weight of the crude gum.

#### 3.3.2. Organoleptic Properties

The colour, taste, surface appearance, odour, shape, and fracture of the crude gum were assessed.

#### 3.3.3. pH of Gum

1.0% w/v aqueous (distilled water) dispersions of *Khaya senegalensis* gum were prepared in distilled water, and the pH was measured using a well-calibrated pH meter (Oakton; Model 1100). Triplicate determinations were carried out, and the mean was recorded.

#### 3.3.4. Moisture Content

Five grams of powdered crude *Khaya senegalensis* gum was weighed precisely into a porcelain crucible that had already been dried to a consistent weight. The gum was set in a hot air oven and kept up at a temperature of 105°C. After 5 hours, the gum was taken out and cooled, after which it was put in a desiccator for 30 minutes. The weight of the crucible together with the gum was recorded. The moisture content or loss on drying was expressed as a percentage of the *Khaya senegalensis* gum sample. The entire process was repeated for the purified gum. The determination was done in triplicate [19].

#### 3.3.5. Total Ash

A clean crucible was placed in an oven until it was dried to a constant weight. It was then taken out of the oven and left in a desiccator to cool. The weight of the empty crucible was recorded. About 2.0 g of the gum sample was placed in the crucible. The sample was then placed into a muffle furnace for 4 hours at a temperature of 550°C. It was allowed to cool below 200°C and maintained for another 20 minutes. The sample was put in a desiccator to cool off completely. The weight of the ashed sample was determined [18].

#### 3.3.6. Solubility

The solubility of khaya gum was assessed in cold and hot distilled water, chloroform, and ethanol (96%). One gram of the gum was placed in 50 mL solvents and permitted to stand overnight. A volume of 25 mL of the supernatants was placed in preweighed Petri dishes and evaporated to dryness over a constant water bath. The mass of the residue with reference to the volume of the solutions was measured using an analytical balance and expressed as the percentage solubility of the gum in the respective solvents [4].

#### 3.3.7. Swelling Index

The swelling index of purified *Khaya senegalensis* gum was ascertained by weighing one gram (1 g) into a 10 mL measuring cylinder. The initial volume of the gum was noted. Distilled water is added to the 10 mL mark. The cylinder was stoppered, mixed lightly, and allowed to stand for 24 hours. The volume occupied by each of the gum sediments were noted after 24 hours [20].

#### 3.3.8. Water Retention Capacity

The content of the measuring cylinder from the previous experiment was filtered with the aid of a calico cloth. The water was completely drained into a dry 10 mL measuring cylinder. The volume of water drained from the gum was recorded, and the difference between the initial volume of mucilage and the volume of water drained was recorded as the water absorbed by the gum sample [20].

#### 3.3.9. Acid-Insoluble Ash

Distilled water was heated to near boiling point; a 5 HCl mixture was prepared by mixing 2 volumes of concentrated HCl with 5 volumes of distilled water. About 25 ml of the 2 + 5 HCl was used to transfer the total ash residue from the crucible to a beaker. The contents of the beaker were stirred with a glass rod and covered with a watch glass. It was then heated gently for about five minutes in a hood. The bottom of the watch glass was rinsed with hot water into the beaker. The acid solution was filtered through an ash filter paper. It was ensured that all traces of the acid and ash were completely rinsed (with hot water) from the beaker and crucible onto the filter. More washing of the filter paper was done until the washings were acid-free to litmus. The filtrate was allowed to drain, and the residue was carefully transferred into a weighed crucible. It was then dried in an oven and ignited in the muffle furnace at 600°C, cooled in a desiccator, and weighed [18].

### 3.4. Effect of Concentration on Viscosity of Mucilage of Purified *Khaya senegalensis* Gum

Various concentrations of *Khaya senegalensis* gum mucilages (5, 10, 20, 30, and 40% w/v) were prepared using distilled water. The viscosity of the prepared samples was measured at a shear rate of 30 rpm using Perten's rapid visco analyser (RVA 4500) at 25°C.

### 3.5. Effect of Temperature on Viscosity of Mucilage of Purified *Khaya senegalensis* Gum

A concentration of 40% w/v mucilage of purified *Khaya senegalensis* gum was prepared using distilled water. At a shear rate of 30 rpm, the prepared sample was determined using Perten's rapid visco analyser (RVA 4500) at temperatures of 25, 50, 65, 75, and 90°C.

#### 3.5.1. FTIR Analysis

FTIR analysis of the gum was carried out using a Fourier transform infrared spectrometer (200-X, SN: 200043). The sample was prepared using distilled water, and the analysis was done by scanning the sample through a wave number range of 400 to 4000 cm^−1^.

#### 3.5.2. Elemental Content Analysis of Purified *Khaya senegalensis* Gum

Inorganic constituents existing in the gum were first removed through the technique of wet washing. 1 g of the sample was weighed into a beaker (250 mL). 10 mL of concentrated hydrochloric acid was introduced, followed by addition of (10 mL) nitric acid, and the beaker was covered using a watch glass. The sample was digested with caution on a hot plate in a fumed chamber, followed by cooling of the solution and addition of 20 mL perchloric acid (70% HClO_4_). The digestion was sustained until the solution appeared clear. After the digestion was done, the solution was cooled a little and then 40+ mL of distilled water was added. The mixture was heated for almost 10 minutes and strained hot into a volumetric flask (100 mL) via a Whatman No. 4 filter paper. The solution was topped to the mark using distilled water. Concentrations of sodium (Na), magnesium (Mg), calcium (Ca), zinc (Zn), copper (Cu), arsenic (As), cadmium (Cd), and lead (Pb) were determined using the atomic absorption spectrophotometer [21].

### 3.6. Determination of Microbial Quality of Purified Gum Mucilage

One percent (1% w/v) of purified *Khaya senegalensis* gum mucilage was prepared with sterile water, and about 0.5 mL of the resultant mucilage was inoculated into a previously sterilised MacConkey agar, mannitol salt agar, cetrimide agar, sabouraud agar, nutrient agar, and bismuth sulphite agar. The inoculated agars were incubated at 37°C for 48 hours, and growth of precise organisms depending on the selective media that were utilised was read as present or absent [22].

#### 3.6.1. Preparation of Emulsions

Calculated amounts of both gum and oil were measured and triturated collectively in a mortar, accompanied by the addition of the calculated volume of water. Upon trituration, the hearing of the clicking sound indicated the formation of the primary emulsion. The primary emulsion formed was transferred to an already calibrated bottle and topped with water to the 100 mL mark. The formulated emulsion was observed for any form of instability [4].

#### 3.6.2. Stabilisation of Emulsion

Castor oil (10 mL) was measured, and 2.50 g of *Khaya senegalensis* gum previously weighed was added and mixed thoroughly with the gum. 5 mL of water was added at once and triturated together until the clicking sound was heard. After the primary emulsion was formed, 0.50% v/v of Tween 80 was added to stabilise the emulsion. Various concentrations of xanthan gum and hydroxypropyl methylcellulose were attempted to stabilise the emulsions, but the desired effects were not achieved. It was then topped up to 100 mL volume with water and firmly covered. Emulsions were observed for creaming and phase inversion.

### 3.7. Effect of pH on Emulsion

The pH of the prepared emulsions was adjusted to 4.0, 5.5, 7.2, 9.0, and 11.0, respectively, using 1M HCl and 1M NaOH solution. The emulsions were stored, and the creaming heights noted.

### 3.8. Effect of Electrolytes on Emulsion

The effect of ionic strength on the stability of the emulsions was determined at various ionic strengths (0.1 M) using NaCl, KCl, and CaCl_2_. The emulsions were stored, and the creaming heights noted.

### 3.9. Creaming Volume Data Comparison

A graph of pH and electrolytes against the creaming volume was plotted to determine how compatible and how stable the emulsions can be when the pH and the concentration of the electrolytes are varied. The points were plotted using GraphPad Prism (version 6). Similarly, the emulsion prepared with *Khaya senegalensis* gum was compared with tragacanth gum. Comparison was done using two-way ANOVA.

## 4. Results and Discussions

### 4.1. Extracted Gum Yield

**Figure 2:**
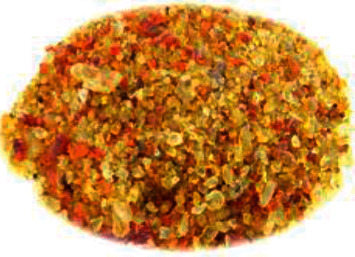
*Khaya senegalensis* gum.

The percentage yield of the extracted gum was 53.33% w/w. It can be inferred from the percentage yield that the purification process was good. Slight modifications of the purification process such as milling of the dried crude gum into coarser particles as shown in Figure 2 and soaking it in four times its volume of distilled water, as well as precipitation of filtered gum solution with four times its volume with 96% ethanol, contributed to this good yield.

### 4.2. Physicochemical Properties

The organoleptic properties were pharmaceutically acceptable, which deems *Khaya senegalensis* gum as a good candidate to be employed as an excipient. From Table 1, the pH of purified *Khaya senegalensis* gum is in the acidic (pH 4.2) range, which is indicative of its pharmaceutical importance. Acidic excipients tend to be the most preferred choice owing to the fact that basic excipients encourage oxidation of sensitive drugs in their formulation.

### 4.3. Solubility of Purified Gum in Different Solvents

From Table 2, it can be observed that there was an increase in solubility of the purified gum in warm water compared with cold water. This could be attributed to the fact that an increase in temperature enhances solubility; thus, solute molecules gain more kinetic energy at elevated temperatures, making them more mobile to interact with solvent molecules. Generally, gums are insoluble in 96% ethanol, a characteristic that is exploited during the precipitation and purification of pure gum from aqueous solutions.

### 4.4. Moisture Content

The moisture content was (11.02% ± 0.500), and it fell within the BP (2013) specifications for biomaterials, the maximum limit is 15% w/w. Due to the fact that moisture induces or speeds up decomposition in the form of microbial growth or hydrolysis, the higher the moisture content, the more the polymer is susceptible to microbial growth. Moreover, 96% ethyl alcohol that was used in the precipitation of the pure gum possesses dehydrating properties and that may have influenced the moisture content of the purified gum.

### 4.5. Ash Values of *Khaya senegalensis* Gum

The total ash of *Khaya senegalensis* gum was 6.033 ± 0.775% w/w. By determining the total ash, the presence of inorganic matter existing in the form of salts such as carbonates, silicates, and phosphates can be quantified. The low total ash value proves its low level of contamination with both plant and siliceous earth materials. Primarily, total ash seeks to detect any form of adulteration, which could be attributed to foreign materials included unknowingly in the cause of the harvesting and purification processes [23]. An accurate way to check for adulteration and the presence of earthly matter is by determining the acid-insoluble ash because the calcium oxide and carbonates yielded by the ignition of oxalate are soluble in HCl when the ash is treated with HCl. The remaining ash (acid-insoluble) is an accurate representation of the earthly matter present [21]. The low figure of acid-insoluble ash (0.45% ± 0.100) indicates that the *Khaya senegalensis* gum contains an insignificant amount of earthly materials. Determination of water-soluble ash aims at identifying constituents which have been extracted with water.

### 4.6. Swelling Index

The swelling index of the gum was (606.667% ± 5.774), which shows that the gum swells six times its original weight. This could be attributed to the highly cross-linked polymer network. The high value of the swelling index obtained for *Khaya senegalensis* gum exhibits its novel excipient potential as diffusion-controlled or erosion-controlled for water-soluble and water-insoluble drugs, respectively. This is because the swelling capacity of a gum demonstrates its hydrophilicity and ability to swell into a gel in an aqueous medium to release an embedded drug.

### 4.7. Effect of Concentration and Temperature on Viscosity of Mucilage of Purified *Khaya senegalensis* Gum

**Figure 3:**
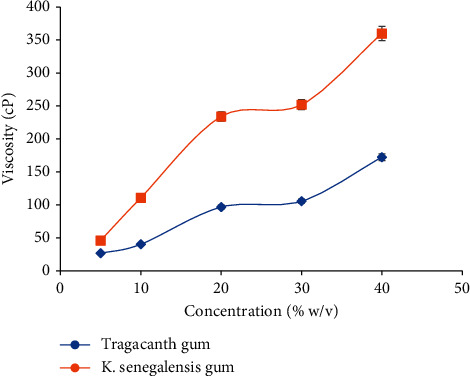
Plot of concentration with respect to viscosity. The error bars represent standard deviations for 5 concentrations in three separate sample runs (*n* = 15).

**Figure 4:**
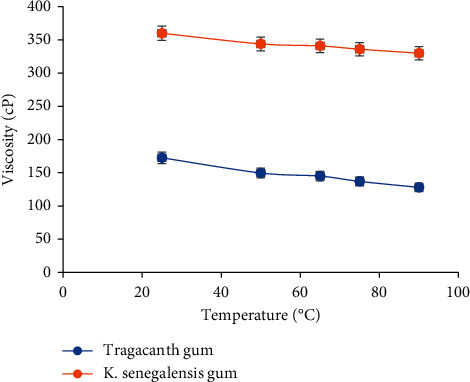
Plot of temperature against viscosity. The error bars represent standard deviations of 1 concentration in three sample runs (*n* = 15).

From Figure 3, it can be observed that the viscosity of *K. senegalensis* gum increased with increasing concentrations. This is of great importance to liquid pharmaceutical preparations such as emulsions and suspensions. As viscosity increases, the rate of creaming or sedimentation decreases and this will result to administering of an accurate dose [4]. The increased rise in viscosity could be attributed to the rapid rise in power of molecule-molecule interaction and the parallel reduction in the molecule-solvent interaction [24]. With the effect of temperature on viscosity results, it can be observed that viscosity decreases as temperature increases as shown in Figure 4. If liquid preparations containing *Khaya senegalensis* gum are stored at elevated temperatures, the consistency of the product may be affected, which could lead to inaccurate dosing. The decrease in viscosity could be attributed to the increase in kinetic energy upon application of heat, which eases the breakdown of intermolecular bonds between contiguous layers [24].

### 4.8. FTIR Analysis

**Figure 5:**
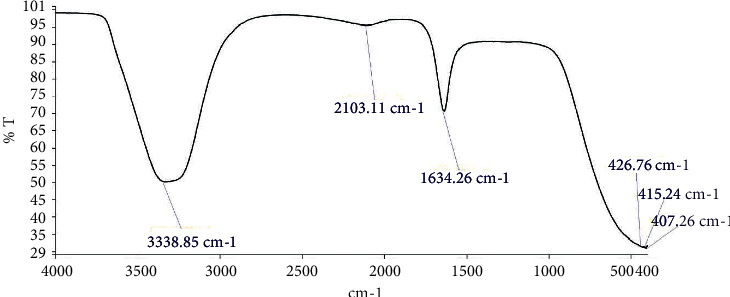
FTIR spectrum of purified *Khaya senegalensis* gum.

The FTIR spectroscopic spectrum of *Khaya senegalensis* gum as shown in Figure 5 indicated intense bands of absorption of functional groups, which were in conformity with literature values and other work findings on *K. senegalensis* gum [16]. Vibrations of stretches and bends identified for *K. senegalensis* gum were specific for the O-H, C≡C, and C=C of the different functional groups. O-H stretches of alcohol, phenol functional groups, occurred at 3338.85 cm^−1^, stretches for the bonds of the alkyne functional groups occurred at 2103.11 cm^−1^, and C=C stretches of alkene functional groups occurred at 1634.26 cm^−1^.

The presence of these diverse functional groups specifies that the parent structure of *Khaya senegalensis* gum is modifiable and can act as a drug carrier for several active pharmaceutical ingredients.

### 4.9. Elemental Analysis

**Figure 6:**
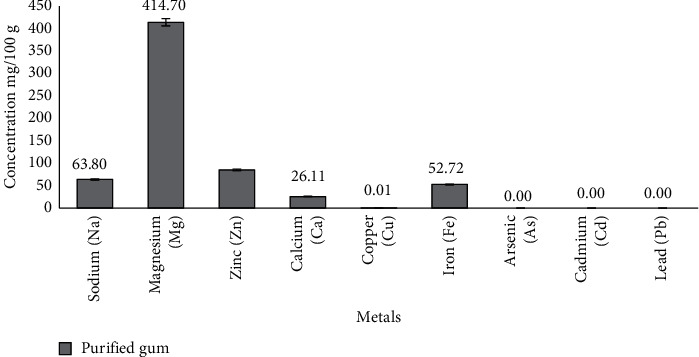
Mineral analysis of purified *Khaya senegalensis* gum.

From the mineral analysis results as shown in Figure 6, the metals present include sodium, magnesium, zinc, calcium, copper, and iron, which were in this order Mg^2+^ > Zn^2+^ > Na^+^ > Fe^2+^ > Ca^2+^ > Cu^2+^. The concentration of copper was found to be within the acceptable limit of 1.3 mg/L per the WHO specification for biomaterials. Again, heavy and toxic metals such as cadmium (Cd), arsenic (As), and lead (Pb) were absent in the purified *Khaya senegalensis* gum. This indicates that *K. senegalensis* gum can be considered a good candidate to be used as a pharmaceutical excipient.

### 4.10. Microbial Quality of Purified *Khaya senegalensis* Gum

Table 3 shows the microbial quality of the purified *Khaya senegalensis* gum. Assessing the microbial load of starting materials and excipients is very critical since it can affect the individual manufacturing phases and in the long run reducing the shelf life of the final product. Therefore, *K. senegalensis* gum intended for use as an emulsifying agent in the formulation of castor oil emulsions for oral use was assessed to know if it was of the right requirement. From the microbial quality results, it can be inferred that the gum was free from objectionable microorganisms such as *E. coli*, *Pseudomonas aeruginosa, Salmonella* spp., and *Staphylococcus aureus.* The total aerobic count (420 cfu/g) was within the acceptable criteria (<10^3^ CFU/g) according to the European Pharmacopoeia [25]. The fungal count was within the accepted range according to the British Pharmacopoeia [22]. This could be attributed to the purification process as 96% ethanol was employed. Most of these objectionable microorganisms would not survive at that condition. This work has shown that purification plays an essential role in gum production as it eliminates debris and certain unwanted materials as well as reduces microbial load.

### 4.11. Formulation of Oil-in-Water Emulsion

In the formulation of oil-in-water emulsion, *K. senegalensis* gum and tragacanth gum (standard) were used as emulsifying agents. Various emulsion stabilisers such as Tween 80 and xanthan gum of varying concentrations were used to improve the stability of the emulsion as shown in Table 5. Double-strength chloroform water was used as a preservative. Castor oil was employed as the dispersed phase. The emulsions were formulated using the dry gum method by employing the 4 : 2 : 1 ratio as shown in Table 4. This method is economical and simple.

### 4.12. Determination of Emulsion Type

To determine the emulsion type, the formulated emulsions were subjected to a dilution test. Thus, the emulsion was diluted with the external phase, in this case which is water. Upon dilution of the emulsion, there was no cracking observed, which infers that the formulated emulsion is an oil-in-water emulsion (O/W). Furthermore, the translucent test spot was employed to affirm the previous test. Here, a drop of the formulated emulsion was smeared onto a piece of paper for 60 minutes. Later, the water smear did not become translucent, but the smear of oil was translucent for a long period of time, leaving spots on the paper. This outcome indicated that indeed the formulated emulsion was O/W. Mostly, the type of emulsifier plays an important role in determining the emulsion type. Emulsifiers with HLB values (hydrophile-lipophile balance) of 8–18 form O/W emulsions [4], in this study which is tragacanth gum.

### 4.13. Droplet Size and Polydispersity Index (PdI) of Emulsions

**Figure 7:**
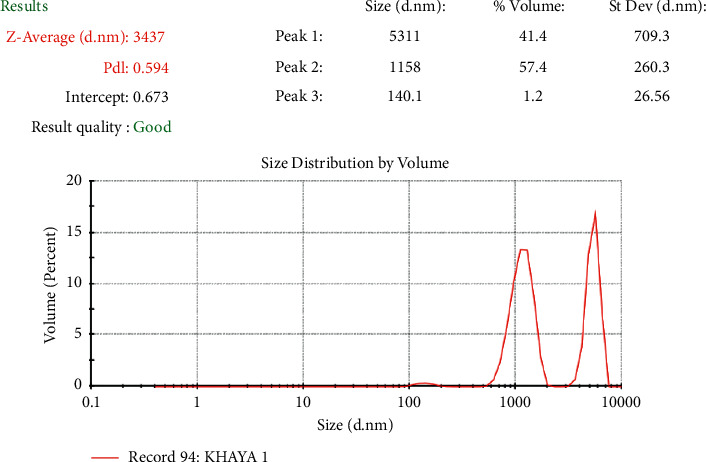
Droplet size and polydispersity index (PdI) of *Khaya senegalensis* gum emulsion.

**Figure 8:**
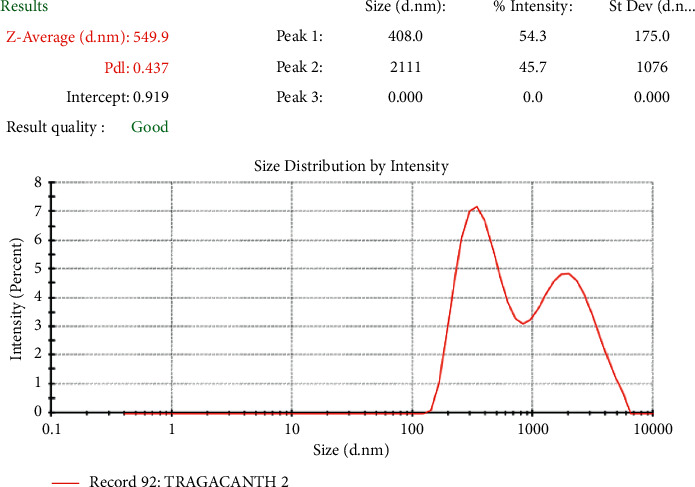
Droplet size and polydispersity index (PdI) of tragacanth gum emulsion.

Droplet size and droplet size distribution are vital variables to consider and are controllable with formulation, raw materials, and the equipment employed to prepare the emulsion [26]. All emulsions prepared with khaya gum and tragacanth gum had a bimodal distribution and droplet sizes within the acceptable range [4]. Bimodal emulsions are characterised by having two different and controlled droplet size distributions. From Figure 7, emulsions prepared with *Khaya senegalensis* gum had an average droplet size of 3.44 *µ*m, whereas those prepared with tragacanth gum had an average droplet size of 0.55 *µ*m as shown in Figure 8. The difference in droplet sizes could be as a result of the difference in particle sizes of the individual emulsifying agents. Reportedly, colloidal systems with lower polydispersity index signify higher stability owing to the lower ripening rate [27]. From the polydispersity analysis, emulsions prepared with tragacanth gum (0.437) were significantly more stable (^∗∗∗∗^*P* < 0.0001, unpaired *t*-test with Welch's correction) than that of the ones prepared with *Khaya senegalensis* gum (0.594).

### 4.14. Zeta Potential Measurement of Emulsions

Electrophoretic measurements of both khaya and tragacanth emulsions were taken. From the zeta potential results, emulsions prepared with khaya gum had a zeta potential of −21 mV, which specifies for threshold of light dispersion. However, emulsions containing tragacanth gum had a zeta potential of −5.15 mV, which is specific for strong agglomeration and precipitation [28]. This decrease in zeta potential in the presence of NaCl can be attributed to compression of the double layer formed around the oil droplets, being similar to observations reported previously in gum arabic and mesquite gum [29]. It can be inferred that emulsions prepared with *Khaya senegalensis* gum was of a higher stability than emulsions containing tragacanth gum.

### 4.15. Effect of pH on Emulsions

**Figure 9:**
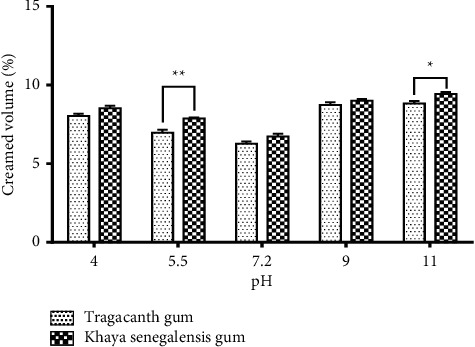
The effect of pH on castor oil emulsion using *Khaya senegalensis* gum as an emulsifier. Values are means ± SEM (*n* = 3). ^∗∗^*P* < 0.01, ^∗^*P* < 0.5 compared with emulsions with tragacanth gum (two-way ANOVA followed by Dunnett's multiple comparisons test).

From the pH results (Figure 9), it can be inferred that both tragacanth and khaya gum performed better in the acidic region than the basic region during the 12 weeks of study. This could be attributed to the reduction in the electrostatic repulsion between the droplets as the pH approached the basic level.

Comparing emulsions prepared with *Khaya senegalensis* gum with those of tragacanth gum, there was a significant difference at pH 5.5, matching their creaming volumes. This result could be attributed to the fact that tragacanth gum forms stable emulsions around that pH. At pH 4, 7.2, and 9, there was no significant difference between *K. senegalensis* gum and tragacanth gum. For pH 5.5 and pH 11, there was a slight difference (*p* < 0.01 and *P* < 0.05, respectively) between the two gums with the tragacanth gum performing better than the *K. senegalensis* gum. Similar results were reported by Farzi et al. [30], who confirmed that tragacanth gum stabilises emulsions by both electrostatic and steric repulsion.

### 4.16. Effect of Electrolytes on Emulsions

**Figure 10:**
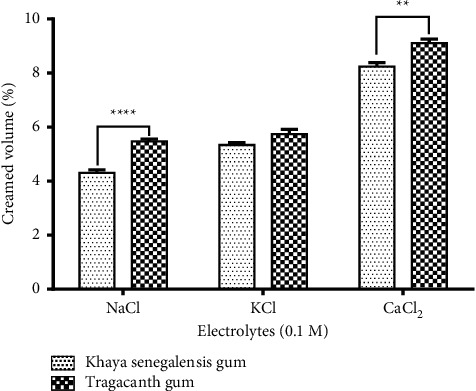
The effect of electrolytes on castor oil emulsion using *Khaya senegalensis* gum as an emulsifier. Values are means ± SEM (*n* = 3). ^∗∗∗∗^*P* < 0.0001, ^∗∗^*P* < 0.01 compared with emulsions with tragacanth gum (two-way ANOVA followed by Dunnett's multiple comparisons test).

Studies have confirmed that salt concentration influences emulsion stability. Electrolytes reduce emulsion stability possibly due to electrostatic screening effect. The effects of 0.1 M concentration of NaCl, KCl, and CaCl_2_ on the emulsions were visually monitored for gravitational phase separation for 12 weeks at 40°C. From the graph (Figure 10), it can be inferred that as the ionic strength of the salts (Na^+^ < K^+^ < Ca^2+^) were increased, creaming also increased. This could be attributed to the fact that ionic strength has a more pronounced effect on the compression of the electric double layer. Emulsions containing *Khaya senegalensis* gum were more compatible and stable than emulsions containing tragacanth gum.

## 5. Conclusion

The *Khaya senegalensis* gum can be purified to improve upon its appearance and quality. The percentage yield obtained from the purification procedure was 53.33%. The gum was free from objectionable microorganisms. The elemental analysis indicated the absence of toxic heavy chemicals like arsenic, lead, and cadmium; this suggests the suitability of the gum to be exploited as a pharmaceutical excipient. Ionic strength has a more pronounced effect on the compression of the electric double layer. Very high or very low pH has an effect on the stability of emulsions.

*Khaya senegalensis* gum can be used as an emulsifying agent with its creaming reduced by addition of low concentrations (0.5%) of Tween 80.

## Figures and Tables

**Table 1:** Physicochemical properties of *Khaya senegalensis* gum.

Parameter	Result
Yield (%)	53.33
Taste	Tasteless
Colour	Yellowish brown to reddish brown
Odour	Odourless
Surface appearance	Glassy and smooth
Shape	Slender and translucent tears
Fracture	Easily fractures
Moisture content (%)	11.02 ± 0.500
pH (1%w/v)	4.2 ± 0.058
Total ash (%w/w)	9.466 ± 0.775
Acid-insoluble ash (%w/w)	0.45 ± 0.100
Water-soluble ash (%w/w)	0.66 ± 0.053
Swelling index (%)	606.667 ± 5.774
Water retention capacity/10 mL	8.167 ± 0.058

**Table 2:** Solubility of purified gum in different solvents (*n* = 3).

Solvent	*Khaya senegalensis* gum (%)
Warm water	0.630 ± 0.061
Cold water	0.427 ± 0.061
Ethanol	0.068 ± 0.018
Chloroform	0.025 ± 0.006

**Table 3:** Microbial quality of purified gum.

Selective medium	Results	Inference
Nutrient agar	Whitish colonies observed	Total aerobic count (420 cfu/g)
Mannitol salt agar	No growth observed	*Staph. aureus* absent
Bismuth sulphite agar	No black colonies observed	*Salmonella* spp. absent
Cetrimide agar	No growth observed	*Pseudomonas* spp. absent
MacConkey agar	No growth observed	*Escherichia coli* absent
Sabouraud agar	Cream colonies observed	Fungal count (<100 cfu/g)

**Table 4:** Stabilisation of emulsions.

Stabiliser	Concentration (%)	Results
Xanthan gum	0.10	Marked creaming
	0.20	Marked creaming
	0.25	Marked creaming
	0.30	Marked creaming
	0.25	Creaming
	1.00	Creaming
	2.00	Creaming
	3.00	Creaming

Tween 80	0.10	Creaming
	0.20	Creaming
	0.30	Reduced creaming
	0.50	Stable

**Table 5:** Stability ratios of castor oil emulsions using *Khaya senegalensis* gum as an emulsifier.

Castor oil	Water	*Khaya senegalensis* gum	Results
25	12.50	6.25	Gum hardened upon addition of water
20	10	5	Cracked
15	7.5	3.75	Cracked
10	5	2.5	Formed and stable
5	2.5	1.25	Formed, less stable

## Data Availability

The data used to support the findings of this study are included within the article.
